# A statistical quality assessment method for longitudinal observations in electronic health record data with an application to the VA million veteran program

**DOI:** 10.1186/s12911-021-01643-2

**Published:** 2021-10-20

**Authors:** Hui Wang, Ilana Belitskaya-Levy, Fan Wu, Jennifer S. Lee, Mei-Chiung Shih, Philip S. Tsao, Ying Lu, on behalf of VA Million Veteran Program

**Affiliations:** 1 Department of Veterans Affairs, Cooperative Studies Program Palo Alto Coordinating Center, 701B North Shoreline Blvd, Mountain View, CA 94043 USA; 2 Department of Medicine, Stanford University School of Medicine, 1265 Welch Road, Stanford, CA 94305-5464 USA; 3 Department of Epidemiology and Population Health, Stanford University School of Medicine, Stanford, CA 94305 USA; 4 Department of Biomedical Data Science, Stanford University School of Medicine, 1265 Welch Road, X359, Stanford, CA 94305-5464 USA

**Keywords:** Data quality assessment (DQA), Electronic health record (EHR), Real world evidence, Clinical informatics, Health care big data, Vital signs

## Abstract

**Background:**

To describe an automated method for assessment of the plausibility of continuous variables collected in the electronic health record (EHR) data for real world evidence research use.

**Methods:**

The most widely used approach in quality assessment (QA) for continuous variables is to detect the implausible numbers using prespecified thresholds. In augmentation to the thresholding method, we developed a score-based method that leverages the longitudinal characteristics of EHR data for detection of the observations inconsistent with the history of a patient. The method was applied to the height and weight data in the EHR from the Million Veteran Program Data from the Veteran’s Healthcare Administration (VHA). A validation study was also conducted.

**Results:**

The receiver operating characteristic (ROC) metrics of the developed method outperforms the widely used thresholding method. It is also demonstrated that different quality assessment methods have a non-ignorable impact on the body mass index (BMI) classification calculated from height and weight data in the VHA’s database.

**Conclusions:**

The score-based method enables automated and scaled detection of the problematic data points in health care big data while allowing the investigators to select the high-quality data based on their need. Leveraging the longitudinal characteristics in EHR will significantly improve the QA performance.

**Supplementary Information:**

The online version contains supplementary material available at 10.1186/s12911-021-01643-2.

## Background

The role of real-world evidence (RWE) is rapidly expanding over the last several years. It is now well recognized that RWE has potential for reshaping clinical research and clinical decision-making, even at regulatory level. As an example, the 21st Century Cures law passed in 2016 requires that FDA considers RWE for supporting regulatory decisions as a means of bringing new treatment to patients more quickly and efficiently. Electronic health records (EHR) data is a major source of RWE as well as a driving force behind RWE use, owing to its big size, rich dimensions, real-time update, and longitudinal characteristics. With the wide-spread adoption of EHRs in the US, the number of research studies based on the EHR data is rapidly increasing. Examples include disease burden [1], post-marketing safety surveillance [2, 3], and comparative effectiveness including synthetic controls [4–6]. However, one major challenge for using the EHR data to support clinical decision making is whether EHR data is of satisfying quality for drawing any meaningful conclusions.

EHRs are routinely collected by providers at a patient care facility for administration use. They are typically not collected to the same standard of quality as those of research data, which are subject to routine monitoring, auditing, and verification. Therefore, before EHR data can be used to answer research questions, it must be assessed for its quality including conformance, completeness, and plausibility [7–11]. The conformance in data quality assessment (DQA) evaluates if data adheres to specified structural and formatting specifications of the database. The completeness examines if the presence or absence of data attributes are within expectation in a database. The plausibility determines the degree to which data values are believable. Plausibility can be further categorized into atemporal plausibility and temporal plausibility. Atemporal plausibility focuses on cross-sectional data features (e.g., height values must be non-negative), while temporal plausibility focuses on a sequence of values over time (e.g., adult height is stable over time). For data as big as the EHR, manual checking is infeasible, and algorithmic methods must be used. There is considerable effort for bringing forth automated quality assessment procedures to screen and clean EHR data [12, 13], but no standard is yet established. The data quality assessment of EHR depends on the EHR system, the protocol of how the data is collected (for example, provider-report vs. self-report), and the types of the data. As an example, methods for cleaning discrete data such as diagnosis codes and continuous data such as weight and height can be quite different. As a result, most DQAs implemented in major data sharing networks are rule-based methods [14, 15]. Rule-based methods are simple to implement but have limited power to detect data issues, especially for temporal plausibility. In this paper, we will introduce a score-based method that addresses the temporal plausibility for continuous measurements in EHR data.

The development of our method is based on the EHR data from the Veterans Health Care Administration (VHA). VHA has the largest integrated federal health care system and formally adopted an EHR system as early as in 1970s. VHA collects complete health-care history of veterans who use its care using VistA, an information technology infrastructure implemented in 1980s. The VistA data is extracted in SAS and SQL and stored in VHA’s corporate data warehouse (CDW). The CDW data since year 2000 is made available to VHA researchers in a structured format. The CDW data is further standardized into the Observational Medical Outcomes Partnership (OMOP) Common Data Model for more efficient use in research [16].

We describe in this article an automated procedure for detection of implausible observations among continuous and autocorrelated variables from the EHR data such as height and weight, and body mass index (BMI) that is derived from these two variables. The algorithm can be applied to other types of continuous data and works best when data can reach a stationary distribution for a reasonable length of time.

## Methods

A widely used method to identify problematic observations in continuous variables is simple thresholding: if a data point falls in an implausible range, it will be considered as erroneous. Thresholding method considers each measurement in isolation and falls into the atemporal plausibility DQA category. However, it ignores the longitudinal profile of a patient in EHR and can result in exclusion of good data or inclusion of erroneous data. For example, a patient weighs consistently of 380 pounds in ten visits in three years. With thresholding method, all the data of this patient is likely considered as error and excluded from analysis. On the other hand, a measurement of 120 pounds for an under-weight patient of 80 pounds is considered good data when it is indeed an error.

The proposed method in this paper addresses the drawbacks of thresholding method. A measurement of a continuous variable is considered of questionable quality if it experiences implausible changes over time. And our statistical procedure calculates a longitudinal plausibility score (Q_R_) based on repeated measurements in that patient.

### Longitudinal plausibility score (Q_R_)

The number of repeated measurements and the time between measurements play important roles in the determination of whether a measurement is plausible or not. Using weight as an example, a patient has 6 weight measurements in 3 months: 5 of them are 200 pounds and one is 180 pounds. The measurement of 180 pounds is likely an error. Another patient has 2 weight measurement one year apart: the first is 200 pounds and the second is 180 pounds. There are likely no errors here.

Considering these factors, we have chosen the exponentially weighted moving average (EWMA) for calculation of the longitudinal plausibility score Q_R_. The rational is two folds. Firstly, EWMA is a commonly used time-weighted method in quality control for manufacturing process due to its ability to model decaying dependencies among data over time. An alternative method also commonly used in quality control process is cumulative sum approach (CUMSUM). But CUMSUM assigns equal weights to every time point and cannot model decaying time dependency in auto-correlated data. Secondly and perhaps more importantly, EWMA is a simple method that can scale to the volume of the data linearly and, therefore, suitable for processing large amount of EHR data.

For an individual patient, suppose that we have a sequence of n measurements $y_{1} ...y_{n}$ taken at time points $t_{1} ...t_{n}$. The EWMA $\overline{y}_{i,EWMA}$ for a measurement *y*_i_ taken at time $t_{i}$, $t_{1} \le t_{i} \le t_{n}$, is defined as a weighted average over the entire sequence:

$$\overline{y}_{i,EWMA} = \frac{{\sum\limits_{j = 1}^{n} {w_{j} y_{j} } }}{{\sum\limits_{j = 1}^{n} {w_{j} } }},$$

where the weight $w_{j}$ is determined by the time interval between $t_{i}$ and each *t*_*j*_: ($1 \le i,j \le n$):

$$w_{j} = \left\{ {\begin{array}{*{20}c} 1 & {{\text{if }}j = i} \\ {e^{{ - \frac{{|t_{j} - t_{i} |}}{\tau }}} } & {{\text{if }}j \ne i} \\ \end{array} } .\right.$$

Parameter *τ* tunes for the dependency of $y_{i,EWMA}$ on its neighboring measurements and affects the smoothness of the EWMA estimates. Larger *τ* leads to more correlated and smoother EWMA estimates.

Let us define $d_{i}$ as the absolute difference between the observed $y_{i}$ and the EWMA estimate:

$$d_{i} = |y_{i} - \overline{y}_{i,EWMA} |$$

The variance estimates of $d_{i}$ is, assuming *y*_*i*_s are independent of each other:

$$Var({d}_{i})=Var({y}_{{t}_{i},EWMA}-{y}_{i})=\mathit{var}(\frac{{\sum }_{i=1}^{n}{w}_{i}{y}_{i}}{{\sum }_{i=1}^{n}{w}_{i}}-{y}_{i})=\left(\frac{{\sum }_{i=1}^{n}{w}_{i}^{2}}{({\sum }_{i=1}^{n}{w}_{i}{)}^{2}}+\frac{2{w}_{i}}{{\sum }_{i=1}^{n}{w}_{i}}+1\right)Var({y}_{i}).$$

We can then derive a Z score for $d_{i}$ and its corresponding two-sided p-value from a standard normal distribution:

$$Z_{R,i} = \frac{{d_{i} }}{{SE(d_{i} )}},{\text{ Q}}_{EWMA,i} = 2(1 - \Phi (Z_{R,i} ))$$

The parameter *τ* tunes the smoothness of moving averages and plays an important role in identifying outliers. We provide a heuristic formula for setting *τ:*

$$\tau = - \frac{\xi /12}{{log(\omega )}},$$

In the formula, *ω* is the desired dependency in percentage a researcher wants to put on the neighboring observations *ξ* month away from *t*. For example, for height, we can assign 90% dependency on observations 1-year (12-month) away from the time point *t* because we expect the adult height to be stable over time. In this case*, ω* = *0.9, ξ* = *12*, and it leads to *τ* = *9.49.* With this *τ*, the dependency on measurements 2-year and 5-year away is 81% and 59%. Meanwhile, the weight measurements have much greater variabilities over time, and it may be more reasonable to assign 90% weight on observations half a month away (i.e. *ξ* = *0.5* and *τ* = *0.4).* The calculation of Q_R_ also requires an estimate of the variance of measurements y_i_ –- Var(y_i_). This variance can be estimated from data using a random effect model. When validation data is available, the tuning parameters *τ* and Var(y_i_) can be searched for to achieve an optimal performance of the algorithm. For height, we used the heuristic formula to choose our tuning parameters. For weight, we optimized *τ* and Var(y_i_) using a validation dataset on the false discovery rate and detection rate (*τ* = *0.5*, Var(y_i_) = 210) (Additional file 1: Methods).

### Thresholding score (Q_S_)

For comparison purpose, we also used thresholding method and computed a QA score Qs for each measurement: the two-sided p-value of a simple Z-score that measures the distance between the observed value y_i_ and the population mean y_u_:

$${Z}_{S,i}=\frac{{y}_{i}-{y}_{u}}{SD({y}_{i})}, \, {\text{Q}}_{S,i}=2(1-\Phi ({Z}_{S,i}))$$

The population mean $y_{u}$ and standard deviations (SD) can be estimated from the reference data (Additional file 1: Methods).

Both Q_R_ and Q_S_ are invariant to the units of the measurements of the outcome. For time measurement in Q_R_, age was used for presentation simplicity. Other time measurements such as calendar years can also be used without changing the results if the time intervals among measurements are preserved. At least two measurements are required for calculation of Q_R_. The more measurements available, the smaller the standard error for EWMA, and hence Q_R_ is more precise.

### Study ethics and participant consent

The Million Veteran Program received ethical and study protocol approval by the Veterans Affairs Central Institutional Review Board and informed consent was obtained for all participants. This methodology study protocol was approved by the Stanford University Institution Research Board. All analyses were based on deidentified data from VA CDW. All methods were carried out in accordance with relevant guidelines and regulations.

## Results

### Demographics and characteristics

We analyzed height and weight data in an MVP (Million Veteran Program) cohort of 496,311 patients using VA EHR data between year 2000 and 2016. A total of 10,960,056 height records and 25,548,357 weight records were analyzed. Most patients in this MVP cohort is male (91.4%), white race (71.6%), and non-Hispanic (89.3%). The median age is 64.4 years old at the enrollment (Additional file 1: Table S1).

For analysis, we required that a height measurement is within the range of 40 and 100 inches and a weight measurement within 40 and 1000 pounds. We also required that at the time a measurement was taken the patient was at least 17 years old in database. We removed height or weight records measured more than 3 times on the same day as these records are likely computer entry errors. These data preprocessing steps resulted in height data of 495,393 patients with 10,945,576 measurements. The weight data included 496,292 patients with 25,400,615 measurements. Among these patients, 485,406 had more than one measurement for height and 493,086 patients for weight. The median of the total number of years of follow-up is 12.2 in the height data and 13.1 in the weight data. The median frequency of measurements is 2 measurements per calendar year for height and 3 for weight (Table 1).

**Table 1:** Characteristics of the height and weight data in the MVP cohort

	Height	Weight
	(N = 495,393)	(N = 496,292)
Total number of records	10,945,576	25,400,615
Number of subjects with multiple measurements, N (%)		
Single measurement	9,987 (2.0%)	3,206 (0.6%)
> = 2 measurements	485,406 (98.0%)	493,086 (99.3%)
> = 3 measurements	472,133 (95.3%)	488,684 (98.5%)
Number of measurements in a subject		
Mean (SD)	22.1 (20.4)	51.2 (59.8)
Median (IQR)	17.0 (8, 29)	38 (19, 68)
Number of years of follow-up for a subject		
Mean (SD)	11.5 (5.2)	12.2 (5.1)
Median	12.2 (7.2, 16.1)	13.1 (7.9, 16.8)
Number of measurements per calendar year in a subject^a^		
Mean (SD)	2.0 (1.2)	3.7 (3.9)
Median	2.0 (1.0, 2.0)	3.0 (2.0, 4.5)

^a^Median is used if subjects have multiple-year data

### Observation-level QA

Our analysis focused on the patients with at least two measurements. Both the longitudinal plausibility score (Q_R_) and the thresholding score (Q_S_) were calculated for each measurement for height and weight.

**Fig. 1:**
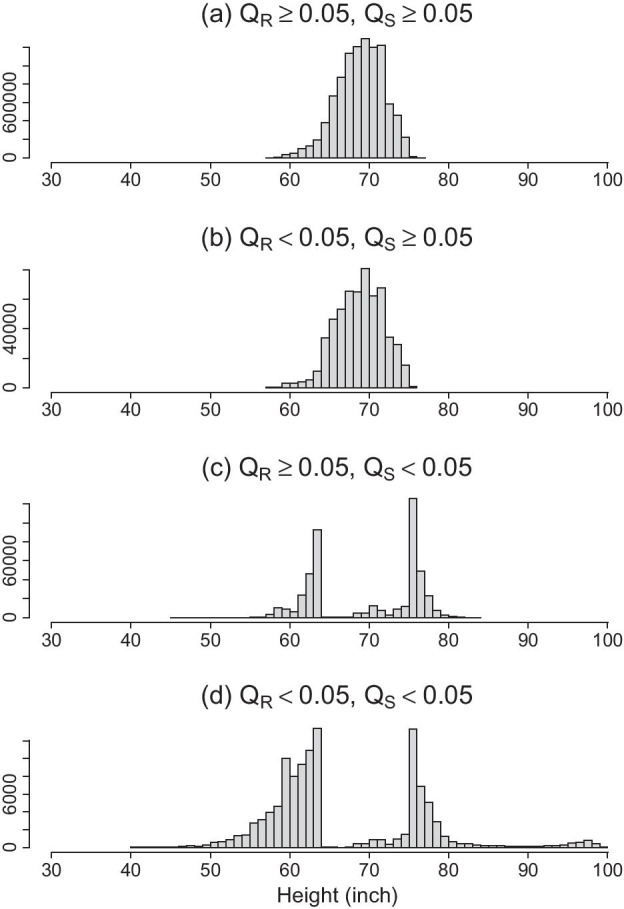
Histograms of height measurements (inch) stratified by the agreements between Q_R_ and Q_S_. **a** Height measurements where both Q_R_ and Q_S_ > 0.05 cutoff value; **b** Height measurements where Q_R_ ≤ 0.05 and Q_S_ > 0.05; **c** Height measurements where Q_R_ > 0.05 and Q_S_ ≤ 0.05; **d** Height measurements where both Q_R_ and Q_S_ ≤ 0.05 cutoff value. X-axis represent the values of height, and y-axis is frequency counts

**Fig. 2:**
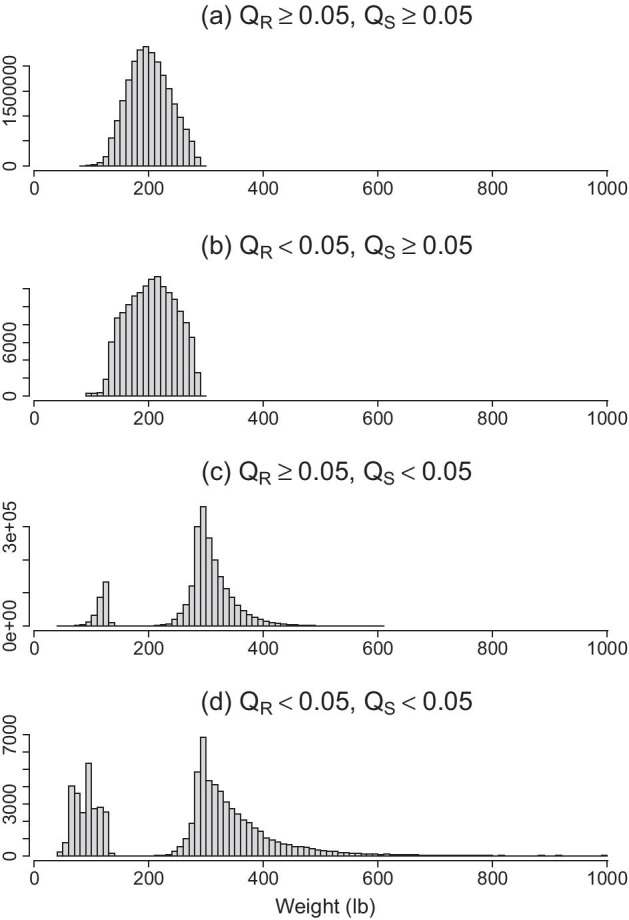
Histograms of weight measurements (lb) stratified by the agreements between Q_R_ and Q_S_. **a** Weight measurements where both Q_R_ and Q_S_ ≥ 0.05 cutoff value; **b** Weight measurements where Q_R_ ≤ 0.05 and Q_S_ ≥ 0.05; **c** Weight measurements where Q_R_ ≥ 0.05 and Q_S_ ≤ 0.05; **d** Weight measurements where both Q_R_ and Q_S_ ≤ 0.05 cutoff value. X-axis represent the values of height, and y-axis is frequency counts

Table 2 compares the proportion of flagged measurements using a cutoff score of 0.05 for both Q_R_ and Q_S_ scores. For height, the two QA scores agreed on 90.5% of the records: 1.0% of data were positive findings and flagged as questionable by both methods, and 89.5% of data were classified as negative and not flagged. The discordance between the two scores counted for 9.5% of the data: 4.2% data were flagged by Q_S_ but considered negative by Q_R_, and 5.3% of the data is vice versa. For weight, only 0.3% data were flagged by both scores; 8.8% data were flagged by Q_S_ while considered negative by Q_R_, while 0.6% data were flagged by Q_R_ but not flagged by Q_S_, and the remaining 90.2% data were in concordance as negatives. The histograms in Figs. 1 and 2 illustrate how different the Q_R_ and the Q_S_ scores can be for identifying problematic data. The observations flagged by Q_R_ (panel b) have a similar distribution as the normal values (panel a), which cannot be identified by Q_S_ scores. In the meantime, The Q_S_ score flagged out-of-range or infrequent values mostly considered negative by Q_R_. Further examination of data suggests that the Q_S_ flags all observations for patients who are over- or under-weight even though these measurements are consistent over time and should be considered accurate (Additional file 1: Fig. S1).

**Table 2:** Proportion of positive and negative measurements identified by Q_R_ and Q_S_

	Positive by Q_R_ (Q_R_ < = 0.05)	Negative by Q_R_ (Q_R_ > 0.05)
Height (N = 10,945,576)		
Positive by Q_S_ (Q_S_ ≤ 0.05)	1.0%	4.2%
Negative by Q_S_ (Q_S_ > 0.05)	5.3%	89.5%
Weight (N = 25,397,409)		
Positive by Q_S_ (Q_S_ ≤ 0.05)	0.3%	8.8%
Negative by Q_S_ (Q_S_ > 0.05)	0.6%	90.2%

### Subject-level QA

A subject-level QA score can be calculated from the Q_R_, defined as the proportion of flagged measurements in a subject. When the proportion of flagged measurements in a patient is too high, all the data of the patient can be considered unreliable and removed from analysis. In our MVP cohort, 9% subjects were identified for having more than 20% of their measurements flagged for height, and 0.47% subjects were identified for weight. These numbers dropped to 2.36% for height and 0.07% for weight when a 50% cutoff was used. More details can be found in Additional file 1: Table S2.

### Validation

To evaluate the performance of the EWMA algorithm, we randomly selected 100 patients whose proportion of flagged measurements were between 0 and 20% using a p-value cutoff of 0.05. The data of these patients were manually reviewed independently by two biostatisticians for identification of problematic measurements. All discrepancies between the two reviewers were reviewed and called independently by a third biostatistician. All reviews were blinded to the results of the algorithm. The incidence of positives is low: for height, 343 out of a total of 6,652 (5%) unique measurements were identified to be problematic in manual review; and for weight, 175 out of 16,825 (1%) unique measurements were considered problematic.

Both the EWMA QA algorithm and the simple thresholding method were evaluated against the manual-review results. False positive rate (FPR), power, and positive and negative predictive values (PPV/NPV) were calculated. The EWMA QA method has an FPR of 1.6% for height and 0.3% for weight. The power of detecting a problematic measurement is 88.9% for height and 75.4% for weight. The PPV (proportion of true positives among called positives) is 75.9% for height and 71.4% for weight. The NPV (proportion of true negatives among called negatives) is 99.4% for height and 99.7% for weight. In contrast, the thresholding method has a much lower power with a higher FPR. The positive predictive values are also much lower than the EWMA method, while the negative predictive values are similar (Table 3). The results are consistent when a cutoff of 0.01 was used for Q_R_ and Q_S_. (Additional file 1: Table S3).

**Table 3:** Validation results

	Longitudinal QA (Q_R_)		Thresholding QA (Q_S_)	
	Height (%)	Weight (%)	Height (%)	Weight (%)
False positive rate (FPR)	1.6	0.3	6.6	12.9
Power	88.9	75.4	17.8	28.0
Positive predictive value (PPV)	75.9	71.4	12.8	2.2
Negative predictive value	99.4	99.7	95.4	99.1

Q_R_ and Q_s_ cutoff = 0.05

### Use case: BMI

BMI is an indicator of obesity and often serves as prognostic factors for a variety of diseases such as diabetes and cardiovascular conditions. The accurate assessment of BMI is required in many studies. With EHR data, we have a way to assess BMI longitudinally. But we want to be mindful of the quality of BMI calculated from height and weight of EHR data. To demonstrate how the quality of height and weight data impacts BMI calculation, we computed BMI in our data using all the data without any QA, data QAed with the thresholding method (Q_S_), and data QAed with the longitudinal method (Q_R_). We then grouped BMIs into four categories of underweight (< 18.5), normal to overweight (≥ 18.5 and < 30), obese class I/II/III (≥ 30), and obese class III (> 40) according to WHO classifications. The proportion of incidence of a patient who ever falls into each BMI class were then calculated. Table 4 compares these proportions among the three methods.

**Table 4:** Proportion of subjects who ever had any BMI in the listed BMI classes

BMI class	All data, No QA^a^ (N = 10,377,511) (%)	Thresholding QA^b^ (N = 9,098,710) (%)	Longitudinal QA^c^ (N = 9,606,933) (%)
Underweight (BMI < 18.5)	4.54	1.47	2.24
Normal to overweight (BMI ≥ 18.5 and < 30)	75.56	76.46	73.07
Obese Class I/II/III (BMI ≥ 30 and < 40)	64.36	61.68	61.06
Obese Class III (BMI ≥ 40)	14.54	6.69	11.39

^a^Excluded 0% data

^b^QA excluded 12.3% data

^c^QA excluded 7.4% data

QA methods excluded all records with a Q_S_ or Q_R_ score ≤ 0.05

N is the number of usable records included in the BMI classification

BMI was calculated when height and weight were measured on the same day. There are 10,377,511 such records in 485,316 subjects. Among these records, 12.32% (1,278,801) were flagged by thresholding QA, and 7.42% (770,578) were flagged by longitudinal QA. The proportion of BMI measures that were obese class III was impacted most by QA methods: 14.54% without QA, 6.69% with thresholding QA, and 11.25% with longitudinal QA. The big differences between thresholding QA and longitudinal QA for extreme obesity illustrates that thresholding QA probably has called many large values as errors while they were not. The proportions in other BMI groups were also affected by QA method but not as greatly.

## Conclusion and discussion

The quality of the EHR data impacts the validity of a study. Therefore, the assessment and the control of the data quality is of the utmost importance in any EHR based research. We have adopted a score-based approach for assessment of the plausibility of the values of continuous variables in EHR data. A quality score is calculated for each observation, and users can select ‘good’ data using user-defined cutoffs based on the need of the study. This is different from the rule-based method where binary calls are made for data quality and users have no control over the QA process. There is always a trade-off between using a strict cutoff and having less data and using a relaxed cutoff but having more errors in data. Researchers also should be mindful about any bias that could be introduced by leaving out any data from an analysis. When necessary, sensitivity analysis may need to be performed using different cutoffs.

In our validation study, the Q_R_ score had 89% power with 14% false discovery rate for height, while for weight, only 75% power was achieved with a 28% false discovery rate. The performance of Q_R_ for weight seems suboptimal although the Q_S_ performs much worse. The reason can be two folds. One is that there is still space to improve the algorithm. For example, the choice of the tuning parameters does impact the performance of the algorithm, and our algorithm also did not model random measurement errors. The other is that many false positives/negatives are borderline cases that are challenging to identify by either an automated algorithm or manual reviews.

Meanwhile, an “implausible” value flagged by the QA method may not necessarily be untrue. For example, a sudden drop of body weight may be related to the change of health condition that itself is an important signal for EHR data mining, while a sudden drop in height may be less plausible. By controlling the number and proportions of flagged records for weight (or BMI), this algorithm may also serve as an option for data science to search clinical events around these records to further understand if they are indeed related to changes of health conditions or no clinical explanations. Still, the unflagged data can reflect the stable status of subjects in the system.

Furthermore, the proposed method is most applicable to detect outliers in stationary data such as weight and height. For non-stationary data on frequent fluctuations (for example, inpatient blood pressure measurements), more advanced methods are needed to derive useful signals for analysis. The longitudinal QA method can also be applied to routine laboratory data such as lipid panels. However, the laboratory data typically have gone through internal QA from the lab, and the added value of using the longitudinal QA is limited. Simple thresholding method is adequate for detecting outliers in the VA lab data. In summary, the effective QA of the EHR data will require a multitude of methods and approaches that can be adapted to specific database and study need. The longitudinal QA method serves as one of the tools in the method toolbox for producing EHR data of research quality.

An alternative approach for handling outliers is to use analytic models less sensitive to outliers and errors in data such as non-parametric statistics and robust regressions. This approach “embeds” outlier handling in the statistical modeling of clinically interesting parameters and hence requires high customization to individual studies. The DQA method, in contrast, identifies problematic data that can be used for many studies and can provide systematic solutions to quality improvements for EHR data. For example, implausible height and weight values may derive a plausible BMI value that is not recognized by roust statistical methods. Nevertheless, robust methods can always be used in addition to DQA methods to improve the quality of the analysis.

As a final remark, we would like to note that the proposed methods detect data errors ad-hoc. Although EHR data are currently being used retrospectively, a better practice is to implement quality control measures in the data collection stage in EHR systems. Simple rule-based checks and prompts such as confirmation of an unexpected value at the input of the data can be much more effective than ad-hoc remedies and save an enormous amount of downstream cleaning work.

## Supplementary Information

**Additional file 1:** Supplement Information.

## Data Availability

The datasets analyzed in this study are currently available to VHA investigators and other approved partners by the Million Veteran Program. Data may become available to non-VA investigators in the future. Please inquire with the VA Million Veteran Program (https://www.research.va.gov/mvp/) for details and updates on access to data.
