# Supplementary material for: A statistical quality assessment method for longitudinal observations in electronic health record data with an application to the VA million veteran program
Source: BMC Med Inform Decis Mak. 2021 Oct 20;21:289. doi: 10.1186/s12911-021-01643-2 (PMC8529838; doi:10.1186/s12911-021-01643-2)
Supplement: Supplementary file 1 — Additional file 1: Supplement Information. [file 12911_2021_1643_MOESM1_ESM.docx]

# **Supplemental Information**

**Supplemental Methods**

Tuning Parameters for Height. The tuning parameters *τ = 9.49* for height was set using the heuristic formula, corresponding to 90% dependency on observations 1-year (12-month) away. The variance of height was estimated as the residual variance from a random effect mode. In the model, the outcome is the height measurements, the covariate is age in years, and the random effect is Subject. The model was performed among subjects who have 10 to 100 measurements. The lmer R package was used to estimate the model.

Tuning Parameters for Weight. The tuning parameters *τ* and Var(y_i_) for weight were optimized using our validation data on the false discovery rate and power. The two parameters were searched simultaneously in the parameter space of 0.4 to 0.8 with incremental step of 0.05 for *τ* and 100 to 300 with incremental step of 10 for Var(y_i_). The value of *τ = 0.5* and Var(y_i_) =210 were selected because they had the most reasonable performance with respect to both the FDR (28%) and power (75%). The figure below plots the FDR against the power for all searched values of parameters *τ* and Var(y_i_).


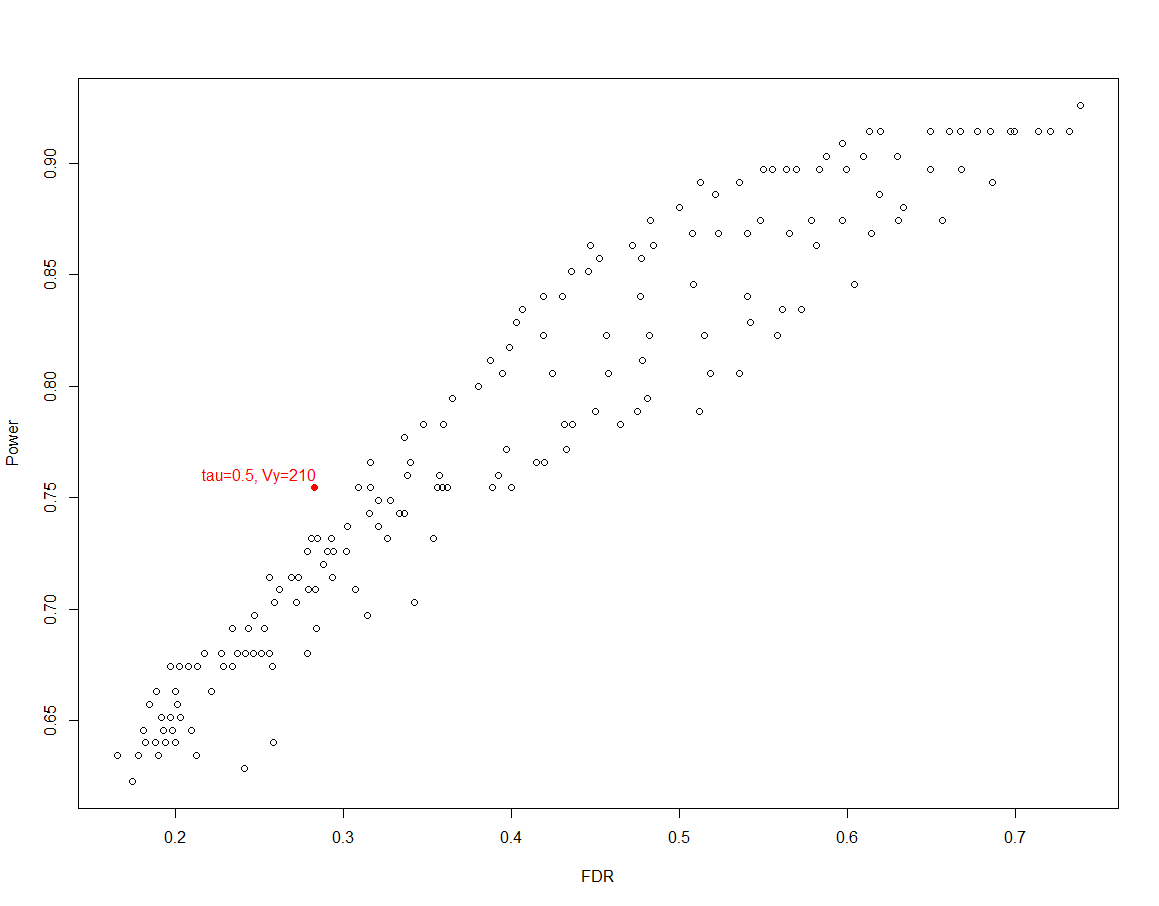


Population Mean Estimates. Population means used in the computation of Q_s_ scores were estimated from a subset of the subjects from our MVP cohort. These subjects were required to have more than 5 measurements over time, and the Q_R_ scores were calculated for all observations. The reference dataset then included the records with a Q_R_ < 0.1 to ensure the quality of the reference data. We then computed among the reference dataset the population median and median absolute deviance (MAD) stratified by sex, ethnicity group, and age group (< 60, 60 – 70, 70 – 80, 80 – 90, >=95). These medians and MAD are used as y_u_ and SE(Y_i_)

**Supplemental Figure 1**. Illustrations of the QA algorithms using individual patient data. Each patient is analyzed using both the thresholding QA score Q_S_ and the longitudinal QA score Q_R_. Black dots represent the observed values; blue crosses represent the moving averages $\overline{y}_{i,EWMA}$ computed from the EWMA; black triangles represent observations flagged by the thresholding QA; and red dots represent observations flagged by the longitudinal QA. The x-axis is time in years, and the y-axis is inches for height and pounds for weight. The labels of x and y axes were removed for protection of patient health information.

1. Height observations from a short patient. All observations except for the first one was flagged by the thresholding QA, yet the longitudinal QA only flagged the first observation. This is a typical example that thresholding QA flags observations that deviate from population mean as erroneous, but these observations are actually correct when assessed longitudinally.
2. Height observations from a patient where thresholding QA and longitudinal QA is completely aligned with each other.
3. Height observations from a patient where the longitudinal QA may have flagged an observation incorrectly (red circle) due to a large shift in data.
4. Weight observations in a patient who was over-weight for a period, and many observations in this period were flagged as erroneous by the thresholding QA.
5. Weight observations in a patient where the thresholding QA and the longitudinal QA are completely aligned with each other.
6. Weight observations in a patient where the longitudinal QA may have falsely flagged an observation due to frequent fluctuations in data.


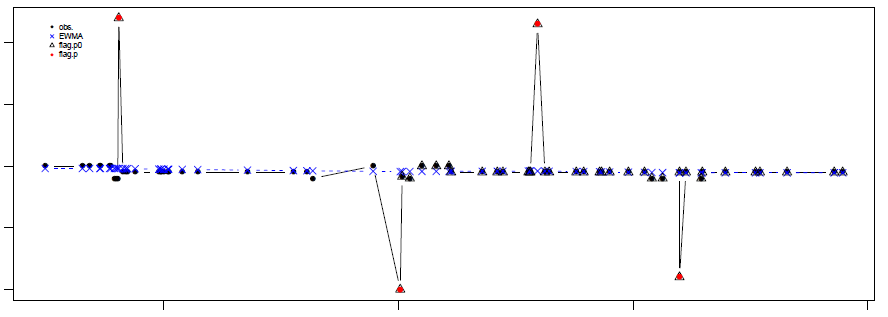


(b)


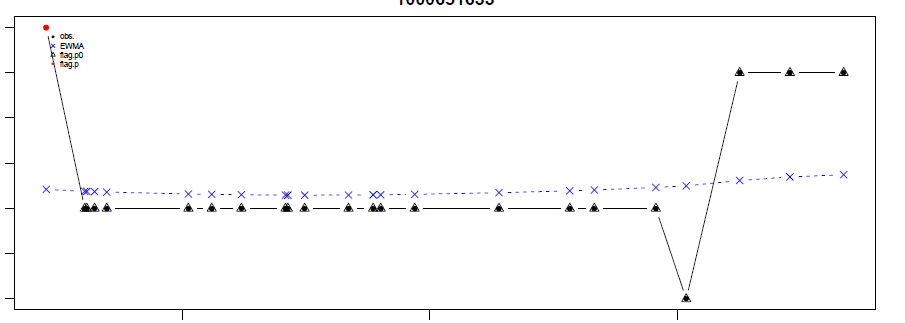


(a)


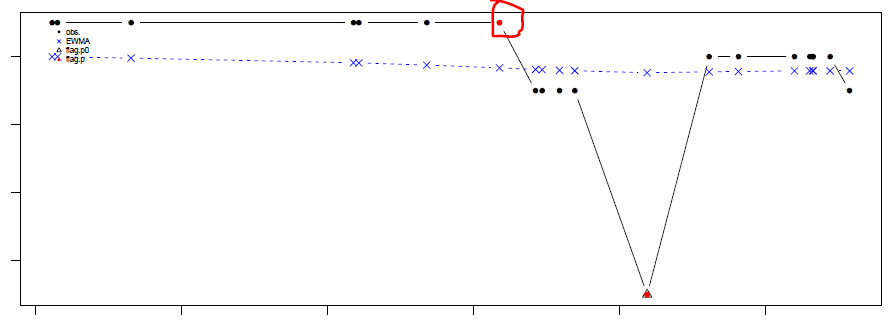


(c)


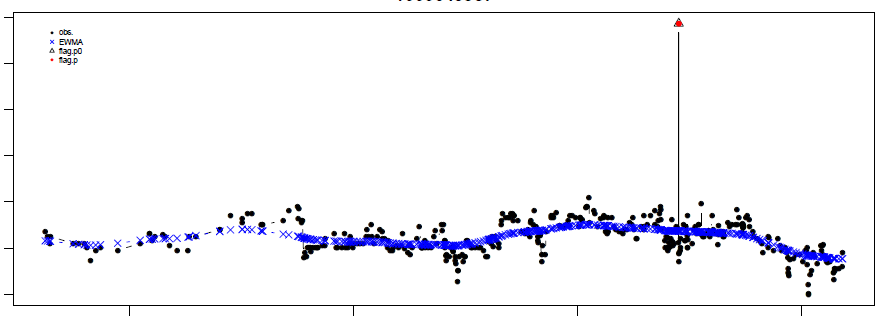

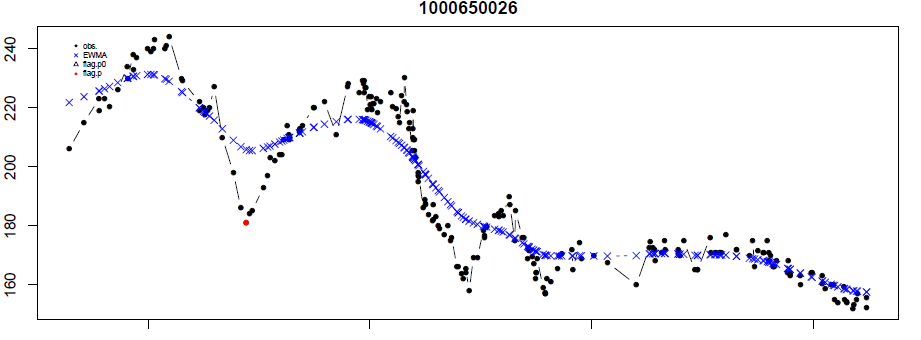


(f)


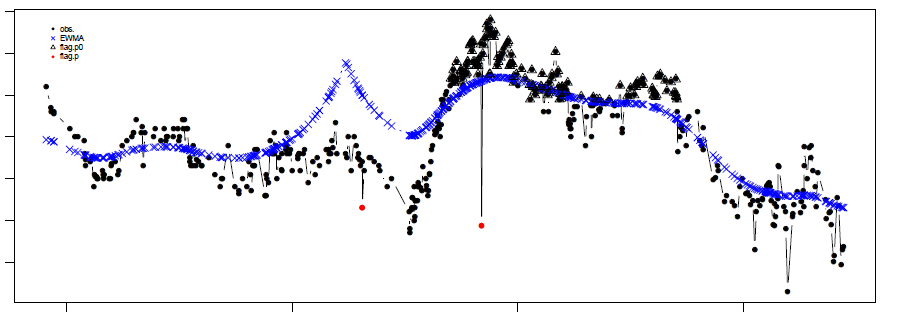


(e)

(d)

**Supplemental Table 1**: Demographics for the MVP patient cohort (N = 496,311).

| Variable | Statistics |
| --- | --- |
|  |  |
| Sex, N (%) |  |
| Male | 453,718 (91.4%) |
| Female | 42,593 (8.6%) |
|  |  |
| Race, N (%) |  |
| White | 355,289 (71.6%) |
| Black or African American | 93,807 (18.9%) |
| Other | 14,560 (2.9%) |
| Unknown | 32,645 (6.6%) |
| Total | 49,6311 (100%) |
|  |  |
| Ethnicity, N (%) |  |
| HISPANIC OR LATINO | 31,336 (6.3%) |
| NOT HISPANIC OR Latino | 443,193 (89.3%) |
| Unknown | 21,782, (4.4%) |
|  |  |
| Age at enrollment |  |
| N | 496,294 |
| Mean (SD) | 62.3 (13.8) |
| Median | 64.4 |
| 1st Q, 3^rd^ Q | 54.5, 70.2 |
| Min, Max | 19.3, 106.1 |

**Supplemental Table 2** Proportion of subjects who have more than 20% or 50% of measurements with p < 0.05.

|  |  | Height  (N=485,406) | Weight  (N=493,086) |
| --- | --- | --- | --- |
| P cutoff = 0.05 | Prop = 0 | 63.09% | 73.06% |
|  | Prop > 20% | 8.94% | 0.47% |
|  | Prop > 50% | 2.36% | 0.07% |
|  | Prop = 100% | 0.71% | 0.02% |
|  |  |  |  |
|  | Mean (SD) | 5.97% (13.87%) | 0.85% (3.23%) |
|  | Median | 0.0% | 0.0% |
|  | 1stQ, 3rdQ | 0.0%, 6.25% | 0.0%, 0.93% |
|  | Min, Max | 0.0%, 100% | 0.0%, 100% |
|  |  |  |  |

**Supplemental Table 3**: Validation Results for Q_R_ and Q_S_ with p-value cutoff of 0.01.

|  | Longitudinal QA (Q_R_) | |  | Thresholding QA (Q_S_) | |
| --- | --- | --- | --- | --- | --- |
|  | Height | Weight |  | Height | Weight |
| False positive rate | 0.4% | 0.09% |  | 0.08% | 4.9% |
| Power | 57.7% | 58.9% |  | 12.5% | 18.3% |
| Positive predictive value  (1-FDR) | 88.0% | 87.3% |  | 89.6% | 3.8% |
| Negative predictive value | 97.7% | 99.6% |  | 95.4% | 99.1% |

Q_R_ and Q_s_ cutoff = 0.01

**EWMA Codes in R**

EWMA2ABS.new <- function(outcome, time, tau){

y <- outcome[!is.na(outcome) & !is.na(time)]

t <- time[!is.na(outcome) & !is.na(time)]

m <- length(y)

if(m > 1){

dtm <- sapply(t, function(ti){abs(ti - t)}) # m x m matrix, column i is t[i] - t

wtm <- exp(-dtm/tau)

wt.sum <- colSums(wtm)

wt.sum <- as.numeric(wt.sum)

ym <- matrix(y, nrow=1, ncol=m)

ywtm <- ym %*% wtm

ywtm <- as.numeric(ywtm)

ewma <- ywtm / wt.sum

c1 <- colSums(wtm^2) / (wt.sum)^2

c2 <- 2 * diag(wtm) / wt.sum

var.coef <- c1 - c2 + 1

}else{

ewma <- var.coef <- wt.sum <- NA

}

return(data.frame(outcome=y, time=t, ewma=ewma, var.coef=var.coef, wt.sum=wt.sum))

}

##########################################################################################################################################################

### ###

### run.ewma: run EWMA2ABS for multiple subjects ###

### ###

### input: ###

### data: a data frame with columns SUBJID, AVAL, and AVISIT ###

### data can not have missing values ###

### tau: EWMA weight ###

### ###

### output: ###

### re: A data frame that combines input data and EWMA ###

### ###

#############################################################################

run.ewma <- function(data, tau){

EWMA <- EWMA.var.coef <- EWMA.wt.sum <- rep(NA, nrow(data))

uid <- unique(data$SUBJID)

for(i in 1:length(uid)){

idx <- which(data$SUBJID == uid[i])

datai <- data[idx,];

r <- EWMA2ABS.new(outcome=datai$AVAL, time=datai$AVISIT, tau=tau)

EWMA[idx] <- r$ewma

EWMA.var.coef[idx] <- r$var.coef

EWMA.wt.sum[idx] <- r$wt.sum

}

re <- data.frame(data, EWMA, EWMA.var.coef, EWMA.wt.sum)

return(re)

}

**VA Million Veteran Program: Core Acknowledgement for Publications**

**Updated December 10, 2020**

**MVP Executive Committee**

- Co-Chair: J. Michael Gaziano, M.D., M.P.H.

VA Boston Healthcare System, 150 S. Huntington Avenue, Boston, MA 02130

- Co-Chair: Sumitra Muralidhar, Ph.D.

US Department of Veterans Affairs, 810 Vermont Avenue NW, Washington, DC 20420

- Rachel Ramoni, D.M.D., Sc.D., Chief VA Research and Development Officer

US Department of Veterans Affairs, 810 Vermont Avenue NW, Washington, DC 20420

- Jean Beckham, Ph.D.

Durham VA Medical Center, 508 Fulton Street, Durham, NC 27705

- Kyong-Mi Chang, M.D.

Philadelphia VA Medical Center, 3900 Woodland Avenue, Philadelphia, PA 19104

- Christopher J. O’Donnell, M.D., M.P.H.

VA Boston Healthcare System, 150 S. Huntington Avenue, Boston, MA 02130

- Philip S. Tsao, Ph.D.

VA Palo Alto Health Care System, 3801 Miranda Avenue, Palo Alto, CA 94304

- James Breeling, M.D., Ex-Officio

US Department of Veterans Affairs, 810 Vermont Avenue NW, Washington, DC 20420

- Grant Huang, Ph.D., Ex-Officio

US Department of Veterans Affairs, 810 Vermont Avenue NW, Washington, DC 20420

- Juan P. Casas, M.D., Ph.D., Ex-Officio

VA Boston Healthcare System, 150 S. Huntington Avenue, Boston, MA 02130

**MVP Program Office**

- Sumitra Muralidhar, Ph.D.

US Department of Veterans Affairs, 810 Vermont Avenue NW, Washington, DC 20420

- Jennifer Moser, Ph.D.

US Department of Veterans Affairs, 810 Vermont Avenue NW, Washington, DC 20420

**MVP Recruitment/Enrollment**

- Recruitment/Enrollment Director/Deputy Director, Boston – Stacey B. Whitbourne, Ph.D.; Jessica V. Brewer, M.P.H.

VA Boston Healthcare System, 150 S. Huntington Avenue, Boston, MA 02130

- MVP Coordinating Centers
  - Clinical Epidemiology Research Center (CERC), West Haven – Mihaela Aslan, Ph.D.

West Haven VA Medical Center, 950 Campbell Avenue, West Haven, CT 06516

- - Cooperative Studies Program Clinical Research Pharmacy Coordinating Center, Albuquerque – Todd Connor, Pharm.D.; Dean P. Argyres, B.S., M.S.

New Mexico VA Health Care System, 1501 San Pedro Drive SE, Albuquerque, NM 87108

- - Genomics Coordinating Center, Palo Alto – Philip S. Tsao, Ph.D.

VA Palo Alto Health Care System, 3801 Miranda Avenue, Palo Alto, CA 94304

- - MVP Boston Coordinating Center, Boston - J. Michael Gaziano, M.D., M.P.H.

VA Boston Healthcare System, 150 S. Huntington Avenue, Boston, MA 02130

- - MVP Information Center, Canandaigua – Brady Stephens, M.S.

Canandaigua VA Medical Center, 400 Fort Hill Avenue, Canandaigua, NY 14424

- VA Central Biorepository, Boston – Mary T. Brophy M.D., M.P.H.; Donald E. Humphries, Ph.D.; Luis E. Selva, Ph.D.

VA Boston Healthcare System, 150 S. Huntington Avenue, Boston, MA 02130

- MVP Informatics, Boston – Nhan Do, M.D.; Shahpoor (Alex) Shayan, M.S.

VA Boston Healthcare System, 150 S. Huntington Avenue, Boston, MA 02130

- MVP Data Operations/Analytics, Boston – Kelly Cho, M.P.H., Ph.D.

VA Boston Healthcare System, 150 S. Huntington Avenue, Boston, MA 02130

- Director of Regulatory Affairs – Lori Churby, B.S.

VA Palo Alto Health Care System, 3801 Miranda Avenue, Palo Alto, CA 94304

**MVP Science**

- Science Operations – Christopher J. O’Donnell, M.D., M.P.H.

VA Boston Healthcare System, 150 S. Huntington Avenue, Boston, MA 02130

- Genomics Core – Christopher J. O’Donnell, M.D., M.P.H.; Saiju Pyarajan Ph.D.

VA Boston Healthcare System, 150 S. Huntington Avenue, Boston, MA 02130

Philip S. Tsao, Ph.D.

VA Palo Alto Health Care System, 3801 Miranda Avenue, Palo Alto, CA 94304

- Data Core – Kelly Cho, M.P.H, Ph.D.

VA Boston Healthcare System, 150 S. Huntington Avenue, Boston, MA 02130

- VA Informatics and Computing Infrastructure (VINCI) – Scott L. DuVall, Ph.D.

VA Salt Lake City Health Care System, 500 Foothill Drive, Salt Lake City, UT 84148

- Data and Computational Sciences – Saiju Pyarajan, Ph.D.

VA Boston Healthcare System, 150 S. Huntington Avenue, Boston, MA 02130

- Statistical Genetics – Elizabeth Hauser, Ph.D.

Durham VA Medical Center, 508 Fulton Street, Durham, NC 27705

Yan Sun, Ph.D.

Atlanta VA Medical Center, 1670 Clairmont Road, Decatur, GA 30033

Hongyu Zhao, Ph.D.

West Haven VA Medical Center, 950 Campbell Avenue, West Haven, CT 06516

**Current MVP Local Site Investigators**

- Atlanta VA Medical Center (Peter Wilson, M.D.)

1670 Clairmont Road, Decatur, GA 30033

- Bay Pines VA Healthcare System (Rachel McArdle, Ph.D.)

10,000 Bay Pines Blvd Bay Pines, FL 33744

- Birmingham VA Medical Center (Louis Dellitalia, M.D.)

700 S. 19th Street, Birmingham AL 35233

- Central Western Massachusetts Healthcare System (Kristin Mattocks, Ph.D., M.P.H.)

421 North Main Street, Leeds, MA 01053

- Cincinnati VA Medical Center (John Harley, M.D., Ph.D.)

3200 Vine Street, Cincinnati, OH 45220

- Clement J. Zablocki VA Medical Center (Jeffrey Whittle, M.D., M.P.H.)

5000 West National Avenue, Milwaukee, WI 53295

- VA Northeast Ohio Healthcare System (Frank Jacono, M.D.)

10701 East Boulevard, Cleveland, OH 44106

- Durham VA Medical Center (Jean Beckham, Ph.D.)

508 Fulton Street, Durham, NC 27705

- Edith Nourse Rogers Memorial Veterans Hospital (John Wells., Ph.D.)

200 Springs Road, Bedford, MA 01730

- Edward Hines, Jr. VA Medical Center (Salvador Gutierrez, M.D.)

5000 South 5th Avenue, Hines, IL 60141

- Veterans Health Care System of the Ozarks (Gretchen Gibson, D.D.S., M.P.H.)

1100 North College Avenue, Fayetteville, AR 72703

- Fargo VA Health Care System (Kimberly Hammer, Ph.D.)

2101 N. Elm, Fargo, ND 58102

- VA Health Care Upstate New York (Laurence Kaminsky, Ph.D.)

113 Holland Avenue, Albany, NY 12208

- New Mexico VA Health Care System (Gerardo Villareal, M.D.)

1501 San Pedro Drive, S.E. Albuquerque, NM 87108

- VA Boston Healthcare System (Scott Kinlay, M.B.B.S., Ph.D.)

150 S. Huntington Avenue, Boston, MA 02130

- VA Western New York Healthcare System (Junzhe Xu, M.D.)

3495 Bailey Avenue, Buffalo, NY 14215-1199

- Ralph H. Johnson VA Medical Center (Mark Hamner, M.D.)

109 Bee Street, Mental Health Research, Charleston, SC 29401

- Columbia VA Health Care System (Roy Mathew, M.D.)

6439 Garners Ferry Road, Columbia, SC 29209

- VA North Texas Health Care System (Sujata Bhushan, M.D.)

4500 S. Lancaster Road, Dallas, TX 75216

- Hampton VA Medical Center (Pran Iruvanti, D.O., Ph.D.)

100 Emancipation Drive, Hampton, VA 23667

- Richmond VA Medical Center (Michael Godschalk, M.D.)

1201 Broad Rock Blvd., Richmond, VA 23249

- Iowa City VA Health Care System (Zuhair Ballas, M.D.)

601 Highway 6 West, Iowa City, IA 52246-2208

- Eastern Oklahoma VA Health Care System (Douglas Ivins, M.D.)

1011 Honor Heights Drive, Muskogee, OK 74401

- James A. Haley Veterans’ Hospital (Stephen Mastorides, M.D.)

13000 Bruce B. Downs Blvd, Tampa, FL 33612

- James H. Quillen VA Medical Center (Jonathan Moorman, M.D., Ph.D.)

Corner of Lamont & Veterans Way, Mountain Home, TN 37684

- John D. Dingell VA Medical Center (Saib Gappy, M.D.)

4646 John R Street, Detroit, MI 48201

- Louisville VA Medical Center (Jon Klein, M.D., Ph.D.)

800 Zorn Avenue, Louisville, KY 40206

- Manchester VA Medical Center (Nora Ratcliffe, M.D.)

718 Smyth Road, Manchester, NH 03104

- Miami VA Health Care System (Hermes Florez, M.D., Ph.D.)

1201 NW 16th Street, 11 GRC, Miami FL 33125

- Michael E. DeBakey VA Medical Center (Olaoluwa Okusaga, M.D.)

2002 Holcombe Blvd, Houston, TX 77030

- Minneapolis VA Health Care System (Maureen Murdoch, M.D., M.P.H.)

One Veterans Drive, Minneapolis, MN 55417

- N. FL/S. GA Veterans Health System (Peruvemba Sriram, M.D.)

1601 SW Archer Road, Gainesville, FL 32608

- Northport VA Medical Center (Shing Shing Yeh, Ph.D., M.D.)

79 Middleville Road, Northport, NY 11768

- Overton Brooks VA Medical Center (Neeraj Tandon, M.D.)

510 East Stoner Ave, Shreveport, LA 71101

- Philadelphia VA Medical Center (Darshana Jhala, M.D.)

3900 Woodland Avenue, Philadelphia, PA 19104

- Phoenix VA Health Care System (Samuel Aguayo, M.D.)

650 E. Indian School Road, Phoenix, AZ 85012

- Portland VA Medical Center (David Cohen, M.D.)

3710 SW U.S. Veterans Hospital Road, Portland, OR 97239

- Providence VA Medical Center (Satish Sharma, M.D.)

830 Chalkstone Avenue, Providence, RI 02908

- Richard Roudebush VA Medical Center (Suthat Liangpunsakul, M.D., M.P.H.)

1481 West 10th Street, Indianapolis, IN 46202

- Salem VA Medical Center (Kris Ann Oursler, M.D.)

1970 Roanoke Blvd, Salem, VA 24153

- San Francisco VA Health Care System (Mary Whooley, M.D.)

4150 Clement Street, San Francisco, CA 94121

- South Texas Veterans Health Care System (Sunil Ahuja, M.D.)

7400 Merton Minter Boulevard, San Antonio, TX 78229

- Southeast Louisiana Veterans Health Care System (Joseph Constans, Ph.D.)

2400 Canal Street, New Orleans, LA 70119

- Southern Arizona VA Health Care System (Paul Meyer, M.D., Ph.D.)

3601 S 6th Avenue, Tucson, AZ 85723

- Sioux Falls VA Health Care System (Jennifer Greco, M.D.)

2501 W 22nd Street, Sioux Falls, SD 57105

- St. Louis VA Health Care System (Michael Rauchman, M.D.)

915 North Grand Blvd, St. Louis, MO 63106

- Syracuse VA Medical Center (Richard Servatius, Ph.D.)

800 Irving Avenue, Syracuse, NY 13210

- VA Eastern Kansas Health Care System (Melinda Gaddy, Ph.D.)

4101 S 4th Street Trafficway, Leavenworth, KS 66048

- VA Greater Los Angeles Health Care System (Agnes Wallbom, M.D., M.S.)

11301 Wilshire Blvd, Los Angeles, CA 90073

- VA Long Beach Healthcare System (Timothy Morgan, M.D.)

5901 East 7th Street Long Beach, CA 90822

- VA Maine Healthcare System (Todd Stapley, D.O.)

1 VA Center, Augusta, ME 04330

- VA New York Harbor Healthcare System (Scott Sherman, M.D., M.P.H.)

423 East 23rd Street, New York, NY 10010

- VA Pacific Islands Health Care System (George Ross, M.D.)

459 Patterson Rd, Honolulu, HI 96819

- VA Palo Alto Health Care System (Philip Tsao, Ph.D.)

3801 Miranda Avenue, Palo Alto, CA 94304-1290

- VA Pittsburgh Health Care System (Patrick Strollo, Jr., M.D.)

University Drive, Pittsburgh, PA 15240

- VA Puget Sound Health Care System (Edward Boyko, M.D.)

1660 S. Columbian Way, Seattle, WA 98108-1597

- VA Salt Lake City Health Care System (Laurence Meyer, M.D., Ph.D.)

500 Foothill Drive, Salt Lake City, UT 84148

- VA San Diego Healthcare System (Samir Gupta, M.D., M.S.C.S.)

3350 La Jolla Village Drive, San Diego, CA 92161

- VA Sierra Nevada Health Care System (Mostaqul Huq, Pharm.D., Ph.D.)

975 Kirman Avenue, Reno, NV 89502

- VA Southern Nevada Healthcare System (Joseph Fayad, M.D.)

6900 North Pecos Road, North Las Vegas, NV 89086

- VA Tennessee Valley Healthcare System (Adriana Hung, M.D., M.P.H.)

1310 24th Avenue, South Nashville, TN 37212

- Washington DC VA Medical Center (Jack Lichy, M.D., Ph.D.)

50 Irving St, Washington, D. C. 20422

- W.G. (Bill) Hefner VA Medical Center (Robin Hurley, M.D.)

1601 Brenner Ave, Salisbury, NC 28144

- White River Junction VA Medical Center (Brooks Robey, M.D.)

163 Veterans Drive, White River Junction, VT 05009

- William S. Middleton Memorial Veterans Hospital (Robert Striker, M.D., Ph.D.)

2500 Overlook Terrace, Madison, WI 53705
